# Evaluating Heat Transfer Conditions in a Plasma-Heated Rotary Kiln for Cement Production

**DOI:** 10.1021/acs.iecr.5c00220

**Published:** 2025-04-10

**Authors:** Alice Fakt, Adrian Gunnarsson, Klas Andersson, Fredrik Normann, Bodil Wilhelmsson

**Affiliations:** † Department of Space, Earth and Environment, Chalmers University of Technology, Hörsalsvägen 7B, SE-412 96 Gothenburg, Sweden; ‡ Heidelberg Materials Cement Sverige AB, Marieviksgatan 25, SE-100 74 Stockholm, Sweden

## Abstract

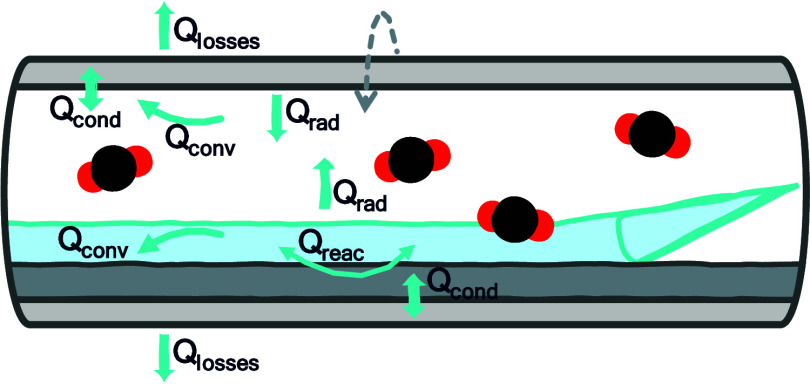

The possibility to use an electrically generated thermal plasma as the heat source in a rotary kiln for cement production is evaluated in this work. In the kiln, the product bed material requires a peak temperature of 1450 °C, which means that the process demands high-temperature conditions. In the conventional process, the overall heat transfer is typically dominated by radiation, with particle radiation from the flame being a major contributor. Thus, the lack of fuel particles in the plasma-heated gas poses a challenge when switching from a flame of solid fuel combustion, resulting in less heat being transferred by radiation and, consequently, a significant change in the overall heat transfer conditions in the kiln. In this work, the heat transfer in a demonstration-scale rotary kiln, heated with an 8-MW_el_ thermal plasma, is modeled. Measurements on a 50 kW_el_ carbon dioxide plasma torch are used to estimate the temperature profile of the plasma. The heat transfer conditions in the kiln, as well as the effects on the heat transfer caused by varying the operational and dimensional parameters are examined within this work. Enhancing the convective heat transfer from the plasma-heated gas can be achieved by tilting the plasma toward the bed, bringing the gas with high temperatures and velocities closer to the bed. The radiative heat transfer can be improved by adding surface area to the gas through particle injection. A combination of tilting the plasma toward the bed and incorporating particles into the plasma-heated gas is found to be the most promising option for increasing the amount of heat transferred to the bed material.

## Introduction

Electrification of fossil-fueled industrial sectors will reduce greenhouse gas (GHG) emissions and mitigate global warming when the electricity comes from nonfossil generation. This work focuses on the cement industry, which accounts for up to 7% of global carbon dioxide emissions.^1^ The production of Portland cement starts with quarried raw material, mainly limestone, which is crushed and mixed with additives to form a meal with the desired material characteristics. The meal is preheated in a preheater tower, using hot exhaust gases from the downstream combustion processes. In the calciner, which is located at the bottom of the preheater tower, heat is supplied from fuel combustion, and about 95% of the limestone in the meal is calcined according to the endothermic reaction R1.
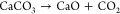
R1

The meal then enters a rotary kiln – a long, rotating, inclined, cylindrical furnace – where it forms a bed and the remainder of the calcination occurs. This is followed by clinkerization as the bed material moves from the higher to the lower end of the kiln. The kiln is equipped with a burner at the lower end, providing heat for the clinker-forming reactions, typically via the combustion of fossil fuels or waste. As the bed moves counter-current to the hot gases, the bed material is heated to about 1450 °C, thereby facilitating the complex solid-phase and liquid-phase chemical reactions that form clinker. The hot clinker is then rapidly cooled, typically on a grate cooler, ensuring the stability of the formed minerals.^2^ Heat from the bed is typically absorbed by an air stream in the cooler, and this stream is used as secondary and tertiary air in the kiln.

About 48% of the heat input to the process is consumed by the endothermic calcination reaction R1.^3^ During calcination, about 60% of the total carbon dioxide emissions from traditional clinker production are inherently formed. The remaining fraction of the carbon dioxide emissions originates from combustion. Therefore, replacing the combustion of fossil fuels with a renewable heat source could reduce the carbon dioxide emissions from the process by up to 40%.

An alternative is to use an electrically generated thermal plasma, employing the carbon dioxide formed during the calcination reaction as the working gas. The feasibility of this strategy is examined in this work. Using carbon dioxide has the advantages that it is available at the plant, is compatible with the cement process, and is a heat-radiating gas. In addition, removing air from the high-temperature process eliminates the formation of nitrogen oxides (NO*_X_*). Furthermore, the use of carbon dioxide as the working gas in the plasma generator generates a flue gas process stream that has a high concentration of carbon dioxide, facilitating an efficient carbon capture process.^2^ However, due to the absence of combustion particles, such as soot, ash, and fuel particles, the radiative heat transfer to the bed material is lower for a thermal plasma than for solid fuel combustion, since carbon dioxide only emits radiation at narrow wavebands in contrast to broadband-emitting particles. As radiation is the dominant heat transfer mechanism in most combustion chambers, especially the rotary kiln,^4^ techniques that enhance heat transfer from the plasma-heated gas to the bed material are needed to ensure an efficient process.

The influences on the heat transfer conditions from varying the dimensional and operational parameters in rotary kilns have been examined extensively. Herz et al.^5^ evaluated the effects on the conductive heat transfer to the bed material of varying the operational parameters and using three different bed materials, and they found that the conductive heat transfer increased at higher rotational speeds and lower filling degree. The effects on the convective heat transfer in an experimental rotary kiln of varying the operational settings were studied by Tscheng et al.,^6^ who concluded that the solids feed rate and kiln inclination had no impacts on the gas-to-wall heat transfer coefficient. A mathematical model for evaluating the radiative, convective, and conductive heat transfer, as well as the regenerative radiative heat transfer in rotary kilns has been developed by Gorog et al.^7^ The impacts on the material flow of different kiln length-to-diameter (L/D) ratios were experimentally evaluated by Chatterjee et al.,^8^ who concluded that for a constant feed rate, the solids’ residence time decreased with increasing kiln diameter. In the work of Proch et al.,^9^ a design for a pilot-scale rotary kiln was developed and the scalability of the design was evaluated. As the kiln design was upscaled for larger bed mass flows, a higher L/D ratio was required if the rotational speed and inclination of the smaller scale kiln were kept constant.

While plasma torch technology is already well-established for applications such as plasma cutting and welding, as well as for the production of high-purity metals,^10^ it is still not used as the single heat source in continuous, high-capacity production units. Nonetheless, according to Rao et al.,^11^ replacing fuel oil burners with plasma torches in chemical and metallurgical processes could reduce the operating costs for existing plants and reduce the capital costs for new plants, while allowing for a significant decrease in GHG emissions. Ko et al.^12^ conducted a numerical analysis to investigate the heat flow variation in a cement rotary kiln equipped with a coal burner in combination with a thermal plasma. The gas temperature distribution in the kiln was found to be more uniform, albeit on average lower, for thermal plasma-assisted combustion, as compared with the case using conventional coal combustion. However, to the best of our knowledge, the size of commercially available plasma generators is today limited to a maximum of 8 MW_el_. Therefore, another challenge for the electrification of the cement process is the scale of the process: with typical production capacities of close to 5000 tonne (t) clinker/day and an average thermal energy demand of 3.5 GJ/t clinker, a fuel demand over 200 MW for a single unit is not uncommon.^13^

This work evaluates the conditions for efficient heat transfer in a plasma-heated rotary kiln used for cement production, by quantifying the heat transferred to the bed material via radiation, conduction, and convection. The influences on the heat transfer of geometrical (kiln length and diameter), and operational parameters (bed material feed rate, plasma torch angle and presence of radiating particles) are evaluated. The work aims to define operational conditions for clinker production, ensuring that the material temperatures required for sintering are reached, in a demonstration-scale rotary kiln, using an 8-MW_el_ CO_2_ thermal plasma torch as the heat source.

## Method and Modeling

**Figure 1:**
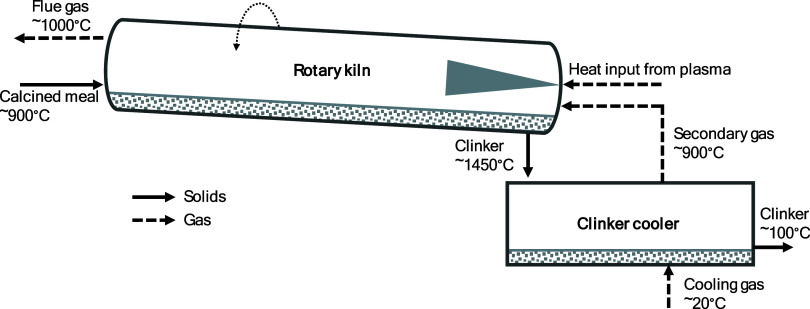
Schematic of the rotary kiln and clinker cooler used in the cement clinker process.

The work is performed as a case study of a demonstration-scale kiln operated with an 8-MW_el_ thermal plasma. The dimensions and operational data are based on an industrial-sized kiln located in Sweden. The carbon dioxide formed from the calcination reaction is recirculated to the clinker cooler and the plasma generator. That is, instead of using ambient air in the cooler, carbon dioxide is used and then supplied to the kiln as secondary gas. The case assumes no false air in the process. Thus, the total gas flow in the kiln comprises the working gas from the plasma generator and the secondary gas supplied to the kiln from the clinker cooler. Figure 1 illustrates the rotary kiln and clinker cooler in the cement clinker process, as studied in this work.

The reference temperature profile of the plasma-heated gas, kiln dimensions, and mass flow rate and inlet temperature of the bed material are varied in the modeling tool to evaluate the impacts on the heat transfer conditions. Finally, a best case is presented in which a high clinker production capacity along with a moderate flue gas temperature is achieved.

### Model Description

**Figure 2:**
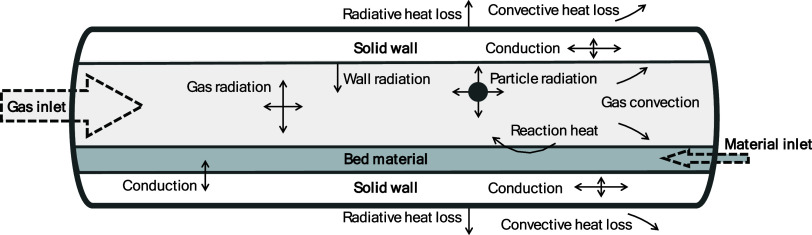
Schematic of a rotary kiln that contains a bed material, showing the heat transfer mechanisms occurring in the kiln.

The applied model to describe the heat transfer in a rotary kiln is based on the work of Gunnarsson et al.^14^ The model has previously been applied to rotary kilns for the production of iron ore pellets. The model solves for the overall heat transfer in the kiln, including heat that is transferred to and from the inner kiln wall and the passing bed material, as well as the outer heat losses at the kiln shell. The focus of the model is on the radiative, convective, and conductive heat transfer in the gas domain. Figure 2 shows a schematic of the rotary kiln, illustrating the heat transfer mechanisms between the gas, bed material, and solid wall.

The model is built around the radiative heat transfer, which is described by the radiative transfer equation (RTE), in which the radiative intensity changes along a set direction *ŝ* due to contributions from emissions, absorption, and scattering. The RTE for a given wavenumber ν is presented in eq 1.
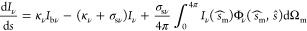
1Where σ_sν_ and κ_ν_ are the scattering and absorption coefficients, respectively, for the present medium,  is the spectral intensity scattered from direction  in direction *ŝ*, *I*_bν_ is the blackbody intensity at the given wavenumber, dΩ_m_ is the solid angle of the small ray in direction , and Φ_ν_ is the scattering phase function. A weighted-sum-of-gray-gases (WSGG) model is used to estimate the radiative gas properties, derived from the work of Ehlmé et al.,^15^ using a set of four gray gases and one clear gas to represent the actual gas mixture in the kiln. The radiative properties of the particles are calculated according to Mie theory, using complex refractive indices from Foster and Howarth.^16^ The modeling tool employs a discrete ordinates method to solve the RTE for a semicylindrical enclosure, in which the bed volume is accounted for, and the kiln volume is divided into cells in the radial, axial and angular directions. Each cell holds parameter values for the temperature, gas concentration, and radiative properties of the present gases and particles.

The particle bed motion is assumed to be of rolling bed mode, which is characterized by a uniform bed level in each cross-section of the kiln. In the modeling tool, the bed is divided into two cell layers, a surface layer and a bottom layer, separated by a fictitious line over which no heat can be transferred. Perfect mixing within a cell is assumed for the surface layer, while no mixing and no slip to the kiln wall is assumed for the bottom layer. The bottom layer is only subjected to conductive heat transfer from the wall, and the surface layer exposed to the flame and passing gas is subjected to convective heat transfer from the passing gas, and radiative heat transfer from the plasma-heated gas as well as the kiln wall. Heat that is being released or absorbed due to reactions occurring in the bed material is accounted for based on the enthalpy of a typical bed material composition, as a function of the temperature, which is estimated using FactSage. The calculations are based on minimizing Gibb’s energy and assume equilibrium conditions. Presented in Table 1 is the bed material composition, representing a typical raw meal mixture for clinker production. Thermodynamic data is based on FactSage databases adapted for clinker production.^17^ At each temperature, the enthalpy of the whole system is calculated, assuming a gas atmosphere typical for clinker kilns. The enthalpy of the bed for each temperature in the range 0–1500 °C is determined by subtracting the enthalpy of the gas from the system’s total enthalpy. The enthalpy data is extrapolated to 2200 °C to allow for higher temperatures in the bed.

**Table 1:** Bed Material Composition used for the FactSage Calculations

compound	w%
Al_2_O_3_	4.32
CaO	65.86
Fe_2_O_3_	2.53
K_2_O	1.20
Na_2_O	0.32
MgO	2.63
SiO_2_	21.22
SO_3_	1.82
Cl	0.08

**Figure 3:**
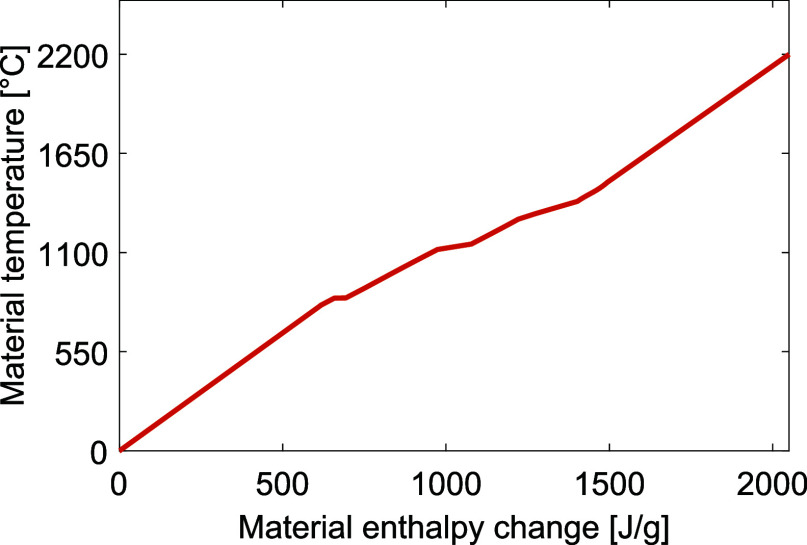
Calculated enthalpy data for the bed material, illustrating the amount of energy that is needed to raise the temperature of the bed material.

The enthalpy data for the bed material used in the model is presented in Figure 3. To ensure sintering, the bed material should reach a temperature of 1450 °C close to the bed outlet, and to avoid backward reactions occurring in the clinker, the material should be rapidly cooled. Thus, achieving sintering temperatures in positions further from the material outlet is not beneficial.

The conductive heat transfer is described by an unsteady-state penetration model, under the assumption that there is no mixing within the cells of the bottom bed layer and no slip to the wall, as has been used previously.^6,18−20^ Penetration models for both the wall and the bed material are used, although no fictitious gas film is considered, according to eq 2, for a short time of *t* seconds.
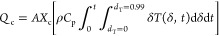
2Here, *X*_c_ is the portion of the bed area *A* that is in direct contact with the wall, *C*_p_ is the specific heat capacity, ρ is the density, and *d*_T_ describes the temperature distribution within the penetration depth, δ, of the bed or wall, which may be found from the Gaussian error function.^14^

The convective heat transfer to the kiln wall (w) and bed (b) material from the hot gases (g) in the kiln are described by the respective heat transfer coefficients, according to the work of Gorog et al.,^7^ as presented in eqs 3 and 4. In this model, the convective heat transfer is only considered in one direction, allowing the wall and bed to be heated or cooled by the gas, while the gas temperature is set as an input parameter in accordance with our previous work.^14^
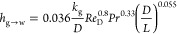
3
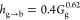
4Here, *D* and *L* are the kiln diameter and length, respectively, *k*_g_ is the thermal conductivity of the gas, and *G*_g_ is the mass flux of gas through the kiln. Radiative and convective heat losses occur on the outside of the kiln as heat is transported through the wall, and this is accounted for in the model.

Given a set of input data, such as gas temperature, gas composition and particle concentration, along with the temperature of the bed at the inlet, the model iteratively calculates the temperatures of the bed and wall from the different contributions of radiation, convection and conduction. Further details of the modeling tool are presented in the paper of Gunnarsson et al.^14^ The overall kiln energy balance is then solved by iteratively updating the flue gas temperature until the total heat demand in the kiln, which comprises the heat transferred to the bed material, heat losses through the kiln wall, and the heat transferred to the gas, is equal to the thermal input provided by the heat source. The procedure has previously been presented by Ehlmé et al.^21^

### Plasma Temperature Profile

Available measurement data for a stand-alone 50 kW_el_ DC, nontransferred, carbon dioxide thermal plasma torch were used to estimate the temperature profile of the 8-MW_el_ plasma torch in this work. Table 2 presents the measurement data-points for the 50 kW_el_ plasma generator, where radiative intensity data were gathered at eight axial positions, *l*_1_–*l*_8_, downstream of the plasma generator. A conical shape was observed for the plasma-heated gas, and the axial and radial temperatures in the gas were estimated based on modeling the radiative intensity for a high-temperature carbon dioxide gas, as described elsewhere.^22^ In Table 2, *T*_*c*,*j*_ corresponds to the center-line temperature for each axial position *l_j_*, *T*_*i*,*j*_ represents the temperatures at each corresponding radial position, *r_i_*, of the plasma-heated gas, and *T*_sur_ represents the temperature of the surrounding gas. The measurements indicate a peak temperature at the apex of the plasma-heated gas, with the temperatures then decreasing both axially and radially as the plasma-heated gas mixes with the surrounding gas. Symmetry around the axis is assumed.

**Table 2:** Representation of the Temperature Profile of the Plasma-Heated Gas, Along with the Corresponding Axial and Radial Measurement Points

	*l*_1_	*l*_2_	*l*_3_	*l*_4_	*l*_5_	*l*_6_	*l*_7_	*l*_8_
*r*_1_	*T*_*c*,1_	*T*_*c*,2_	*T*_c,3_	*T*_*c*,4_	*T*_*c*,5_	*T*_*c*,6_	*T*_*c*,7_	*T*_*c*,8_
*r*_2_	*T*_2,1_	*T*_2,2_	*T*_2,3_	*T*_2,4_	*T*_2,5_	*T*_2,6_	*T*_2,7_	*T*_2,8_
*r*_3_	*T*_3,1_	*T*_3,2_	*T*_3,3_	*T*_3,4_	*T*_3,5_	*T*_3,6_	*T*_3,7_	*T*_3,8_
*r*_4_	*T*_sur_	*T*_4,2_	*T*_4,3_	*T*_4,4_	*T*_4,5_	*T*_4,6_	*T*_4,7_	*T*_4,8_
*r*_5_	*T*_sur_	*T*_sur_	*T*_sur_	*T*_5,4_	*T*_5,5_	*T*_5,6_	*T*_5,7_	*T*_5,8_
*r*_6_	*T*_sur_	*T*_sur_	*T*_sur_	*T*_sur_	*T*_6,5_	*T*_6,6_	*T*_6,7_	*T*_6,8_
*r*_7_	*T*_sur_	*T*_sur_	*T*_sur_	*T*_sur_	*T*_sur_	*T*_7,6_	*T*_7,7_	*T*_7,8_
*r*_8_	*T*_sur_	*T*_sur_	*T*_sur_	*T*_sur_	*T*_sur_	*T*_sur_	*T*_8,7_	*T*_8,8_
*r*_9_	*T*_sur_	*T*_sur_	*T*_sur_	*T*_sur_	*T*_sur_	*T*_sur_	*T*_sur_	*T*_9,8_

The upscaled 8-MW_el_ plasma is assumed to preserve the conical shape, applying a constant velocity scaling method to derive the base radius of the cone, *r*_9_, at the downstream axial *l*_8_-position. With a constant length-to-radius ratio from the 50 kW_el_ plasma, the length of the 8-MW_el_ plasma, *l*_8_, could be calculated. To express the temperature profile of the 50 kW_el_ plasma, correlations describing the decreases in the radial and axial temperatures from the peak temperature, *T*_*c*,1_, were established. Based on the measurement data, the temperature at the *l*_8_-position of the center-line (*T*_*c*,8_) was found to be approximately 72% of the peak temperature, and the temperature along the center-line was found to be accurately described by a linear correlation, showing good agreement with the measurements. To express the radial temperature decrease from the center-line, a temperature ratio, *x*_*i*,*j*_, was introduced, relating the radial temperature decrease within the heated gas cone for each axial position to the radial temperature decrease to the surrounding gas, as expressed by eq 5:
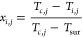
5

**Figure 4:**
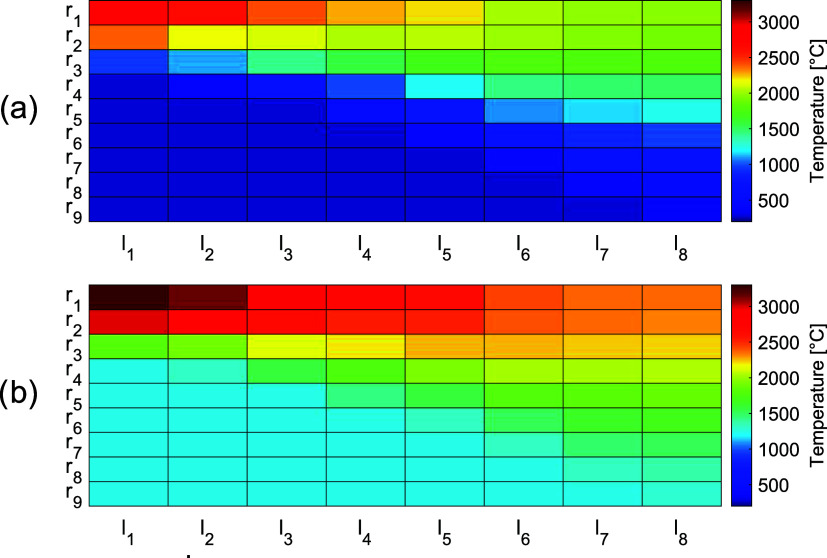
(a) Temperature profile of the 50 kW_el_ plasma-heated gas, where *r*_9_ corresponds to a radius of 0.02 m and *l*_8_ is a length of 0.14 m. (b) The upscaled temperature profile of the 8-MW_el_ plasma-heated gas, where the values of *r*_9_ and *l*_8_ are 0.28 and 1.8 m, respectively.

When scaling up the temperature profile from 50 kW_el_ to 8 MW_el_, the derived temperature correlations are assumed to still apply at each position in the plasma-heated gas, thereby allowing the temperature profile of the plasma to be estimated from a maximum temperature and the temperature of the surrounding gas. Figure 4a represents the temperature profile of the 50 kW_el_ plasma-heated gas, and Figure 4b shows the conical approximation and the temperature profile of the upscaled 8-MW_el_ plasma-heated gas. The peak temperature for the 8-MW_el_ plasma is estimated from the enthalpy of the gas, at a position close to the plasma generator, based on the electrical input and the gas flow through the plasma generator.

The temperature profile of the tilted plasma torch is estimated by radially displacing the horizontal temperature profile. From a set tilt angle, the number of cells occupied by the tilted plasma is calculated, and the temperature profile is shifted downward until the center line of the plasma meets the bed. Important to note is that the cell volume is lower for cells in the center of the cylindrical furnace, hence tilting the plasma will cause the higher temperatures in the center of the plasma to occupy a slightly larger volume. The temperature profile is linearly interpolated from the point where the plasma cone meets the bed to the gas outlet.

To evaluate the sensitivity to the assumed temperature field, the peak temperature and the spread (length and radius) of the plasma-heated gas were varied within ± 20% of the reference value.

### Kiln Dimensions

The dimensions of the demonstration-scale rotary kiln are based on those of an industrial unit located in Sweden. The dimensions are scaled to maintain the L/D ratio, as well as the axial gas velocities at the kiln inlet. As the starting point for the initial dimensioning of the kiln, a production rate of 10 t/h of clinker is set. The volumetric flow of carbon dioxide, supplied to the kiln from the cooler, is estimated based on the cooling requirement of the clinker in the cooler.

To examine the effects on the heat transfer of varying geometrical and operational parameters, the temperature profile dimensions of the kiln were varied according to Table 3. Dimensional parameters include variations of the kiln length and radius, and the feed rate of the bed material. Having the means to improve the heat transfer from the plasma gas to the bed material, the effects of tilting the plasma torch toward the bed and adding particles to the gas were examined. Parameter value ranges were expanded (upward and downward) in accordance with modeling results, until there was no longer any noticeable impact on the heat transfer conditions.

**Table 3:** Set of Varied Parameters and Reference Case Dimensions

parameter	value range	refs case	unit
kiln length	12–30	18	m
kiln radius	0.3–0.8	0.48	m
material feed rate	7–30	10	t/h
plasma tilt angle	0–35	0	°
particle mass flow	0–465	0	kg/h

At the material inlet, the bed is assumed to hold a temperature of 900 °C, as it would in the industrial process where it is fed to the kiln from the calciner. The secondary gas introduced into the kiln is set to hold a temperature of 980 °C, in accordance with the temperature conditions of the clinker cooler in the industrial process, and it is set to maintain a constant temperature until it mixes with the plasma-heated gas further inside the kiln. In the conventional process, the flue gas exits the kiln at a temperature of up to 1200 °C; for retrofitting and energy optimization purposes, a similar temperature is desired. Higher flue gas temperatures would indicate inefficient heat transfer from the plasma-heated gas to the bed material, and would entail a risk of damaging the downstream process equipment.

## Results and Discussion

The calculated temperature profile for the reference kiln is presented as a temperature map, being the input when varying the dimensions and other operational parameters of the kiln. The initially calculated set of dimensions, referred to as the reference case, is presented and discussed, followed by the effects on the temperature of the bed material and flue gas for each altered parameter. In addition, for a varying feed rate, tilted plasma, and particle addition, the influences on the radiative, convective and conductive heat transfers are presented and discussed. Lastly, a suggested set of operational parameters is presented, for which the material reaches the required temperatures with a moderate flue gas temperature.

### Reference Case

**Figure 5:**
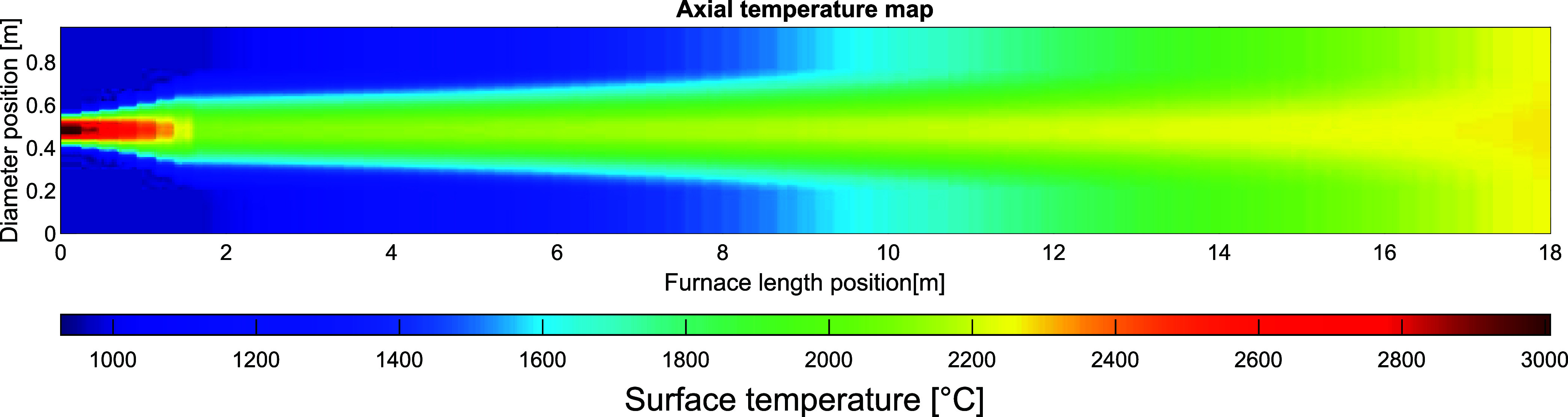
Temperature map of a vertical cross-section of the kiln, showing the gas in the kiln with the plasma torch located to the left.

Figure 5 shows the resulting temperature profile of the plasma-heated gas for the 8-MW_el_ plasma torch. The scaling of the conical plasma results in a plasma-heated gas having a radius of 0.28 m, at a distance of 1.8 m downstream of the plasma generator. At a position close to the plasma generator, the plasma-heated gas holds a calculated maximum temperature of 3007 °C with the axial and radial gas temperature profiles in accordance with the temperature map shown in Figure 5. The temperature profile is linearly interpolated from the axial end of the conical plasma, to the gas outlet, which is set to maintain a uniform temperature at the axial outlet position.

Using a feed rate of 10 t/h of material, the bed and flue gas outlet temperatures reached 1390 and 2275 °C, respectively, indicating inefficient heat transfer from the plasma-heated gas to the bed material. The resulting heat balance is presented in Table 4, showing that most of the supplied heat stays within the gas rather than being transferred to the bed material.

**Table 4:** Heat Balance Results for the Reference Case

heat transfer	[MW]
heat absorbed by bed material	1.74
*radiation*	0.83
*convection*	0.35
*conduction*	0.57
heat absorbed by flue gases	6.06
outer heat losses	0.12
total heat demand in the kiln	7.92

Varying the peak temperature and the dimensions of the plasma-heated gas had no significant impacts on the heat transfer conditions for the reference kiln dimensions, achieving values for the bed and flue gas temperatures that were close to the reference case. This indicates that a slight modification in the scaling of the temperature profile and conical approximation of the plasma-heated gas does not substantially influence the heat transfer conditions in the kiln.

### Kiln Length

**Figure 6:**
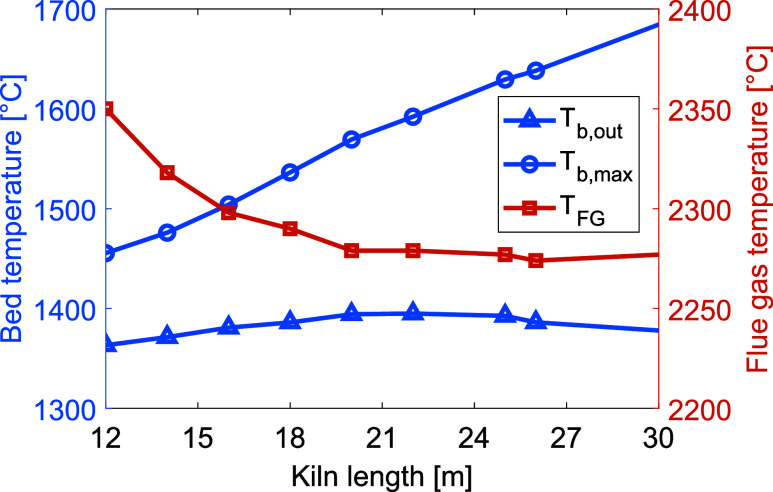
Temperatures of the bed material (left *y*-axis) and flue gas (right *y*-axis) in the kiln with increasing kiln length. To highlight the relevant data range, the axes do not start from zero.

Figure 6 shows that varying the kiln length has impacts on the bed material peak and outlet temperatures, as well as on the flue gas temperature. An increased kiln length results in an increased peak temperature of the bed material, while only small variations in the bed outlet temperature are seen. As indicated by the reduction in flue gas temperature and increased peak bed temperature, the heat transfer to the bed is increased for a longer kiln. The solids residence time is increased as the length of the kiln increases, allowing the bed more time to absorb heat from the hot gas. It should be noted that for a kiln length over 20 m, there is no increase in the bed outlet temperature, indicating that a longer kiln will not increase the material temperature at the outlet, even though the peak material temperature is increased.

Although the temperature of the material at the outlet increases slightly, the bed temperature increase is not sufficient to reach the sintering temperature at the bed material outlet. As shown in Figure 6, the peak temperature of the bed material and the temperature at the material outlet temperature differ, risking backward clinker reactions due to the bed being cooled at a slow rate at the outlet. This issue is most likely attributable to the temperature of the secondary gas being set so as to maintain a constant temperature until mixed with the plasma-heated gas, thereby cooling the bed near the material outlet. While a reduction in the flue gas temperature is apparent, the temperature is still significantly higher than the desired level, suggesting that just increasing the length of the kiln is insufficient.

### Kiln Radius

**Figure 7:**
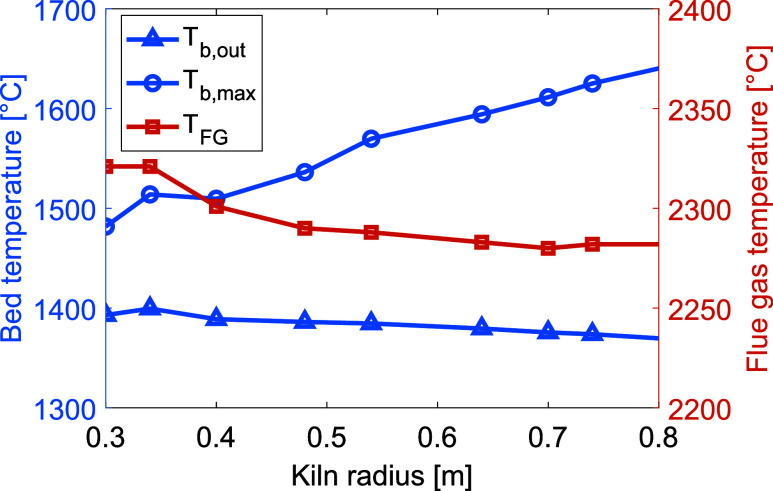
Temperatures of the bed material (left *y*-axis) and flue gas (right *y*-axis) for increasing kiln radius. To highlight the relevant data range, the axes do not start from zero.

With respect to increasing the kiln radius, results similar to those obtained for increasing the kiln length were observed. The resulting temperatures for the bed material and flue gas are presented in Figure 7. When retaining the material filling degree of the kiln, the bed heat transfer area increases with the radius, as does the bed residence time, resulting in increases in the peak temperatures of the bed, while the material outlet maintains a more or less constant temperature. Sintering temperatures are reached but not in proximity to the outlet, again running the risk of backward reactions. The gas velocity is reduced for a larger radius due to the larger cross-sectional area, causing a reduction in convective heat transfer, although this seems to have little impact on the total heat transfer to the bed.

Overall, only a small reduction in flue gas temperature from the smallest radius to the largest was observed, indicating that increasing the kiln dimensions is not sufficient for enhancing heat transfer from the plasma-heated gas to the bed material.

### Material Feed Rate

**Figure 8:**
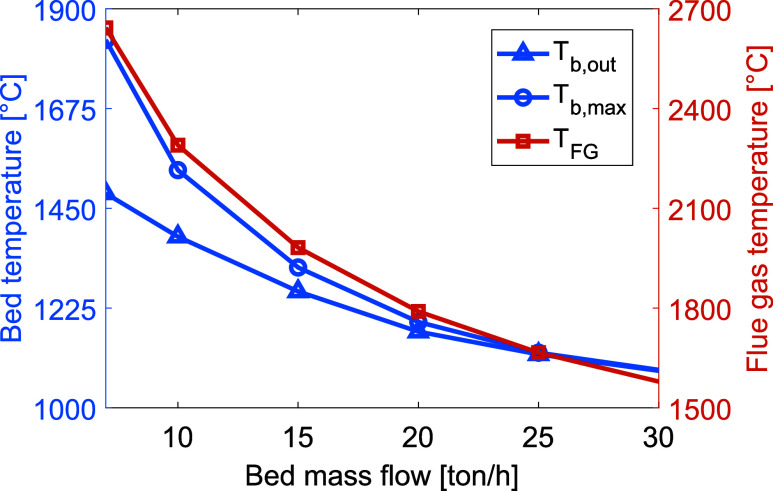
Temperatures of the bed material (left *y*-axis) and flue gas (right *y*-axis) for increasing production rates. To highlight the relevant data range, the axes do not start from zero.

Presented in Figure 8 are the bed and flue gas temperatures for increasing mass flow. The bed material acts as a heat sink in the process, such that a higher feed rate increases the heat demand in the kiln. Thus, an increase in the bed mass flow causes an effective reduction of the flue gas temperature, but also a decreased bed outlet temperature, since the heat input is kept constant. As the filling degree is kept constant, the lower material temperatures can also be attributed to the reduced residence times for increasing feed rates, which does not allow the material enough time to absorb the heat. Reducing the material mass flow to 7 t/h allowed the bed material to reach sintering temperatures, albeit with a flue gas temperature of 2940 °C. Thus, simply reducing the material mass flow is not an efficient measure for improving the heat transfer conditions in the kiln.

**Figure 9:**
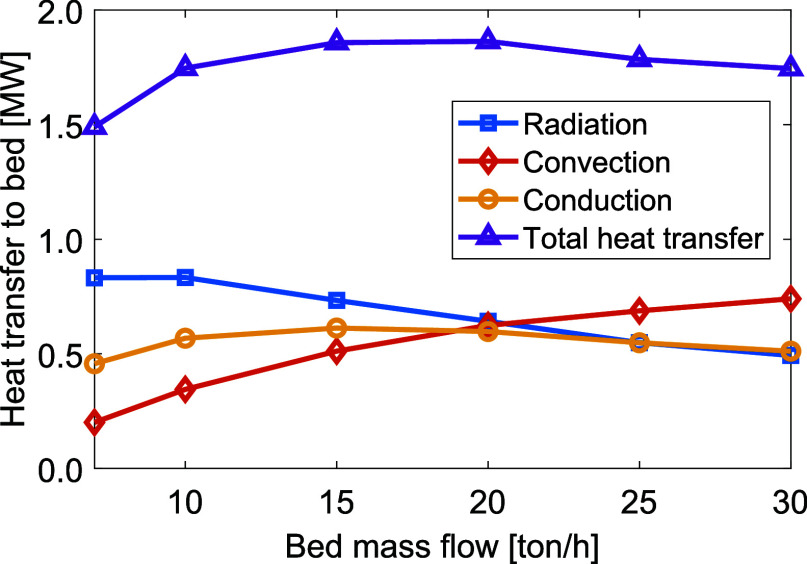
Levels of heat transferred to the bed via radiation, convection, and conduction with increasing production rates.

As presented in Figure 9, both the radiative and conductive heat transfer to the bed are reduced, while the convective heat transfer is increased for increasing mass flows. It should also be noted that the total heat transfer to the bed is increased for mass flows up to 20 t/h. For higher mass flows, the temperature of the bed decreases, which leads to a larger difference in temperature between the gas and solids. As a result, the convective heat transfer increases at positions close to the plasma generator. Due to the lower bed and gas temperatures at higher mass flows, the kiln wall is cooled, thereby reducing the radiative and conductive heat transfer from the wall to the bed. For lower mass flows, the radiative heat transfer is the dominating mechanism. However, when the production rate exceeds 20 t/h the convective heat transfer surpasses the radiative heat transfer, due to the larger difference in average temperature between the gas and solids. The residence time of the bed material is drastically reduced as the material feed rate is increased, which likely causes the reduction in total heat transfer to the bed.

### Plasma Tilt Angle

**Figure 10:**
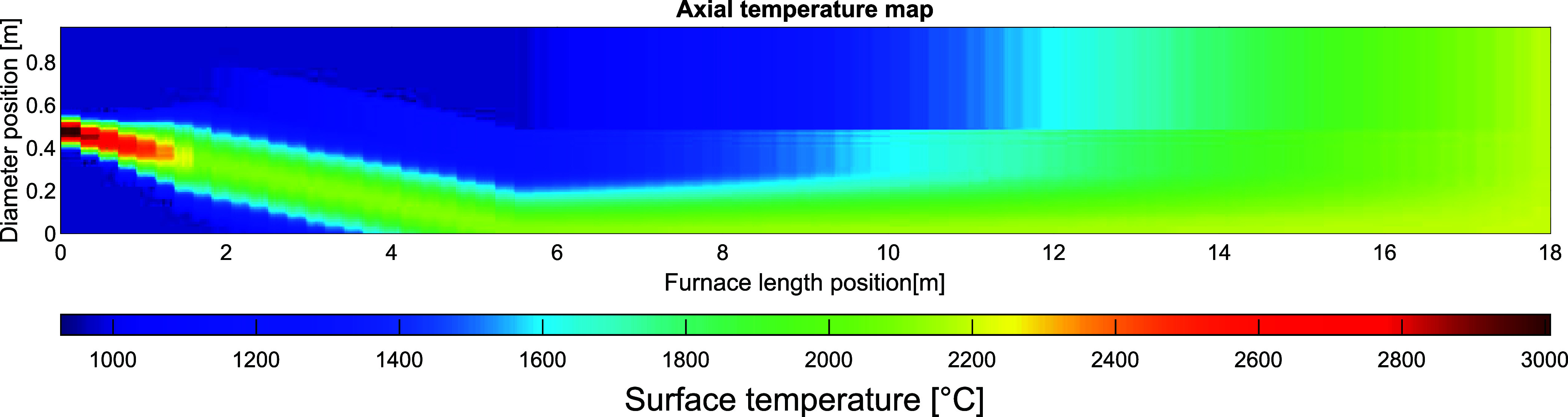
Temperature map of a vertical cross-section of the kiln, showing the temperature profile of the gas with the tilted plasma torch located to the left.

Tilting the plasma torch from the center-line at an angle toward the bed brings the high temperatures and high velocities of the plasma-heated gas closer to the surface of the bed. Figure 10 presents a temperature map of the gas in the vertical cross-section of the kiln, where the plasma has been tilted 5° from the center-line. The maximum temperature of the plasma gas and the temperature of the secondary gas remain constant. However, due to the increased heat transfer to the bed, the temperature at the gas outlet is reduced to 2193 °C, as compared with the reference case shown in Figure 5.

**Figure 11:**
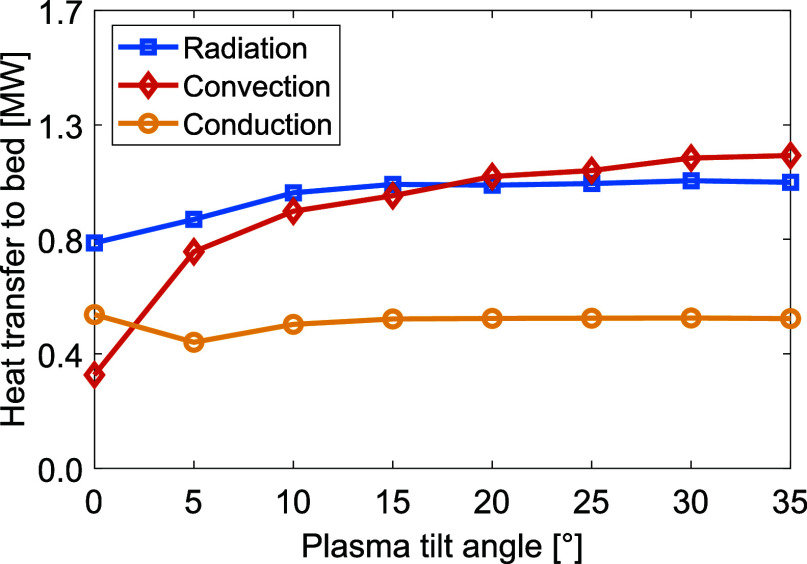
Levels of heat transferred to the bed via radiation, convection, and conduction for different plasma torch tilt angles.

**Figure 12:**
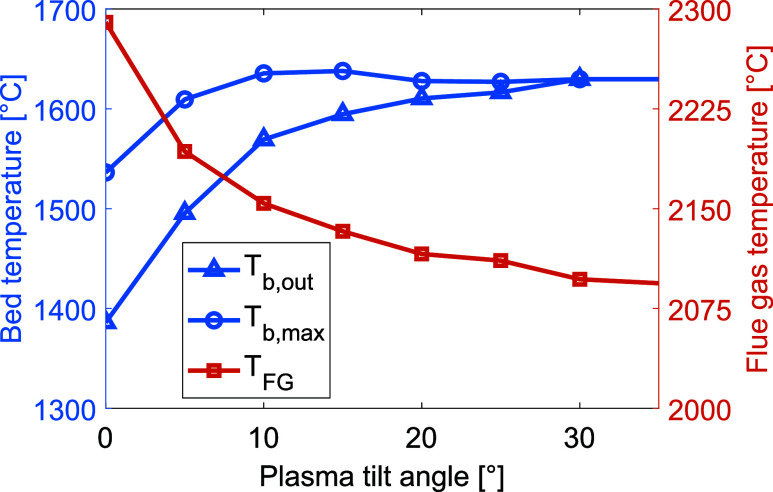
Bed material temperature (left *y*-axis) and flue gas temperature (right *y*-axis) for varying plasma torch tilt angles. To highlight the relevant data range, the axes do not start from zero.

Figure 11 shows the levels of heat transferred to the bed via radiation, convection, and conduction for varying tilt angles and a bed feed rate of 10 t/h. Tilting the plasma torch results in substantial increases in the radiative and convective heat transfers, while the conductive heat transfer remains close to constant. Due to the increased gas temperature and gas velocities at the bed surface, both the radiative heat transfer and convective heat transfer to the bed increase. The total heat transferred to the bed increases from 1.74 MW for the nontilted plasma, to 2.76 MW for the plasma torch tilted at an angle of 35° to the bed. This indicates that most of the heat is still lost with the gases, as also indicated by the high flue gas temperatures in the range of 2000–2200 °C, as shown in Figure 12.

The temperatures of the bed and flue gas for varying tilt angles of the plasma torch are presented in Figure 12, revealing that only a slight tilt of around 5° is needed for the material to reach a sintering temperature. Tilting the plasma further than 30° has little effect on the heat transfer, and the peak material temperature and outlet temperature are seen to converge. While a reduction of the flue gas temperature is evident, it remains significantly higher than that seen in a combustion-based system.

### Projected Surface Area of Particles

**Figure 13:**
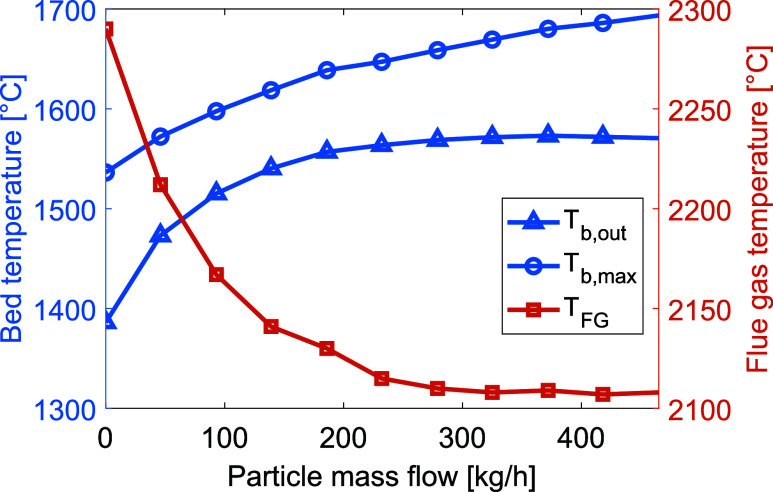
Temperatures of the bed material (left *y*-axis) and flue gas (right *y*-axis) with increasing particle content. To highlight the relevant data range, the axes do not start from zero.

In the model, inert, radiating particles were added to the plasma-heated gas in terms projected surface area per hour, but are here converted to a raw meal mass flow rate. Spherical particles are assumed, having an average radius of 78 μm and density 2700 kg/m^3^. The particles enhance the radiative heat transfer from the plasma-heated gas to the bed material, promoting an increase in the product temperature. The particles are distributed along the geometrical dimensions of the plasma gas, being introduced at a position close to the plasma generator and following the conical shape of the plasma-heated gas. Figure 13 presents the temperatures of the bed material and flue gas for increasing the particle content of the plasma gas.

**Figure 14:**
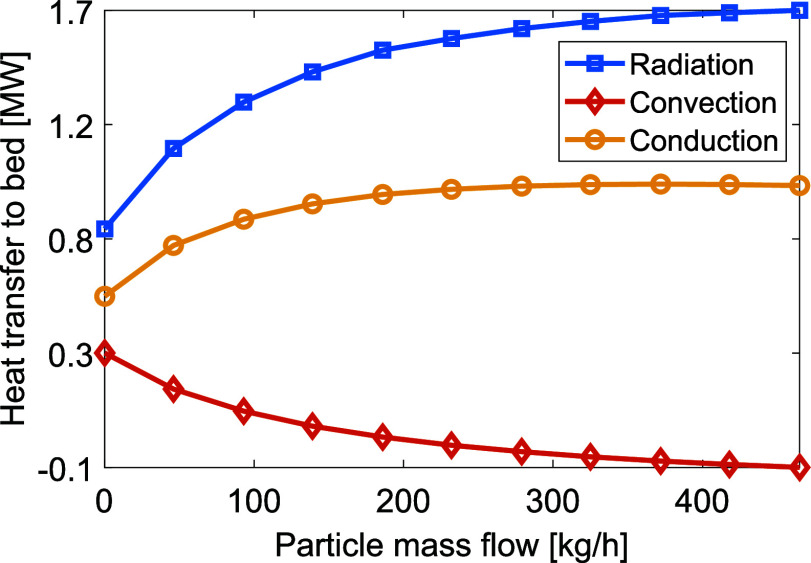
Levels of heat transfer to the bed via radiation, convection, and conduction for increasing particle content of the plasma gas. Note that the *y*-axis does not start from zero, as the values become negative for high levels of particle addition.

It appears that a particle feed of 50 kg/h is sufficient to achieve clinkerization temperatures at the material outlet. For reference, an 8 MW_th_ coal flame would have a corresponding particle mass flow of approximately 983 kg/h, for a typical coal used in rotary kiln applications having a maximum projected area of 153 m^2^/kg.^23^ The respective contributions to heat transfer from the radiation, convection, and conduction processes for various particle contents are shown in Figure 14. When the particle content is increased, radiation becomes the dominant heat transfer mechanism, with convection becoming negligible (or even negative) at a particle content of 230 kg/h, at which point the secondary gas starts to cool the bed. It is noteworthy that the conductive heat transfer increases significantly with the addition of particles, due to increased radiative heat transfer to the wall from the heated gas, which in turn increases the heat transfer from the wall to the bed via conduction.

### Suggested Operational Parameters

As the results indicate, a combination of tilting the plasma torch toward the bed and adding particles is necessary to achieve adequate heat transfer conditions, along with adjusting the material mass flow to reduce the temperature of the flue gas. A suggested set of parameters for which the bed reaches the sintering temperature, along with a moderate flue gas temperature, is presented in Table 5.

**Table 5:** Set of Suggested Kiln Parameters

parameter	suggested parameters	unit
kiln length	18	m
kiln radius	0.48	m
material feed rate	25	t/h
plasma tilt angle	30	°
particle mass flow	255	kg/h

**Figure 15:**
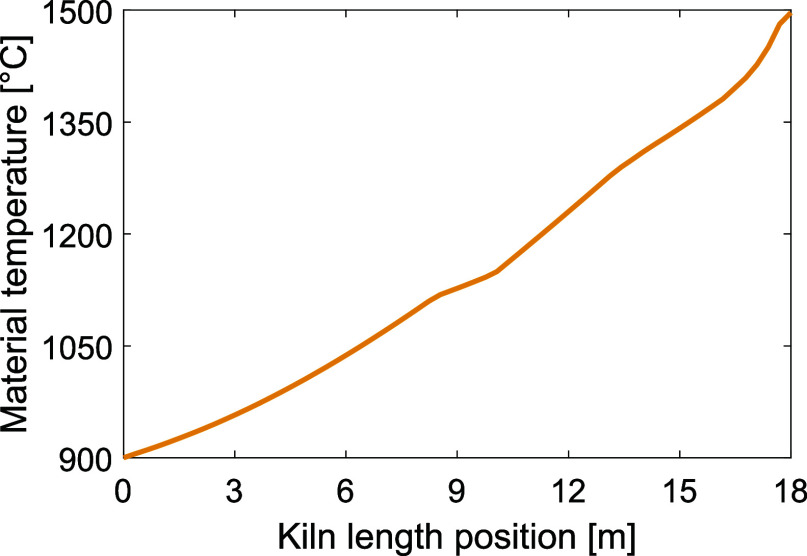
Temperature of the bed material in the kiln, with the material inlet located at length position 0. Note that the *y*-axis starts from the bed inlet temperature at 900 °C.

Figure 15 presents the resulting temperature profile of the bed material in the kiln, for a material mass flow of 25 t/h. For the same length and radius as the reference case but with a tilted plasma torch, added particles, and an increased mass flow rate, sintering temperatures are reached at the material outlet along with a flue gas temperature that is close to 1200 °C.

Compared to the reference case, for which the flue gas and the material at the bed outlet held temperatures of 2275 and 1390 °C, respectively, the flue gas temperature is reduced to 1216 °C while still allowing the bed to reach 1496 °C at the bed outlet. The energy balance for this case is presented in Table 6, where approximately 73% of the total heat input is transferred to the bed, as compared with the reference case (see Table 4), in which the total heat transferred to the bed corresponds to around 22% of the total heat input to the kiln. This indicates the strong impact on the heat transfer of the combination of tilting the plasma torch and particle addition.

**Table 6:** Heat Balance Results for the Suggested Operational Parameters

heat transfer	[MW]
heat absorbed by bed material	5.74
*radiation*	3.18
*convection*	1.42
*conduction*	1.14
heat absorbed by flue gases	2.06
outer heat losses	0.10
total heat demand in the kiln	7.90

## Conclusions

Within this study, the impacts on the heat transfer conditions of changing the geometrical dimensions of the kiln, as well as the operational parameters, which included the bed mass flow, tilting the plasma toward the bed, and adding particles to the plasma-heated gas, have been evaluated. In addition, a set of dimensions and operational parameters is presented that ensures efficient heat transfer from the plasma-heated gas to the bed.

The results of this study highlight the possibility to use a thermal plasma torch with carbon dioxide as the carrier gas to produce cement clinker in a rotary kiln. However, a nonangled, particle-free plasma is unable to achieve the desired heat transfer and bed temperatures, regardless of kiln dimensions. Tilting the plasma toward the bed, to increase convective heat transfer, or injecting particles into the plasma to increase radiative heat transfer are possible strategies to achieve the desired clinkerization temperatures. As the two techniques improve different heat transfer mechanisms, it is efficient to combine them. Increasing the bed mass flow, thereby increasing the heat demand in the kiln, is beneficial in terms of reducing the flue gas temperature. The convective heat transfer is found to be primarily contributing to the total heat transfer to the bed material, mainly due to the high-to-extreme temperatures of the plasma-heated gas and the lack of radiating particles.

With the focus of this work being on the impact on the heat transfer conditions in the kiln, the effect on the solids’ motion when varying the operational conditions of the kiln were not considered. The filling degree is assumed to remain constant when changing the kiln dimensions. However, altering the kiln radius, length, and material feed rate will affect the bed motion and the effect on the filling degree, so this should be considered in future work. Due to the extreme conditions in the plasma-heated gas, the lack of qualitative data on the temperature distribution raises uncertainty regarding the validity of the approximation of the temperature profile in this work, requiring further validation.
